# 3D Printing Soft Matters and Applications: A Review

**DOI:** 10.3390/ijms23073790

**Published:** 2022-03-30

**Authors:** Shuai Zhan, Amy X. Y. Guo, Shan Cecilia Cao, Na Liu

**Affiliations:** 1 Materials Genome Institute, Shanghai University, Shanghai 200444, China; zzzzzs@shu.edu.cn (S.Z.); guoxiaoyun@shu.edu.cn (A.X.Y.G.); 2 Department of Materials Science and Engineering, University of California, Berkeley, CA 94720, USA; 3 School of Mechatronic Engineering and Automation, Shanghai University, Shanghai 200444, China

**Keywords:** additive manufacturing, soft materials, bionics, soft robotics, flexible electronics, biomedical engineering

## Abstract

The evolution of nature created delicate structures and organisms. With the advancement of technology, especially the rise of additive manufacturing, bionics has gradually become a popular research field. Recently, researchers have concentrated on soft robotics, which can mimic the complex movements of animals by allowing continuous and often responsive local deformations. These properties give soft robots advantages in terms of integration and control with human tissue. The rise of additive manufacturing technologies and soft matters makes the fabrication of soft robots with complex functions such as bending, twisting, intricate 3D motion, grasping, and stretching possible. In this paper, the advantages and disadvantages of the additive manufacturing process, including fused deposition modeling, direct ink writing, inkjet printing, stereolithography, and selective laser sintering, are discussed. The applications of 3D printed soft matter in bionics, soft robotics, flexible electronics, and biomedical engineering are reviewed.

## 1. Introduction

Macromolecules composed of numerous repeating subunits are called polymers, which are similar to biomaterials, such as hydrogels [1], silicone elastomers [2], and polycaprolactone (PCL) [3], and play a key role in applications with biological interfaces, including soft robotics [4,5], flexible electronics [6], and biomedical engineering.

3D printing, which is also called additive manufacturing, has become a popular technique to fabricate complex 3D structural matters from various materials, such as metals, ceramics, and polymers [7]. Unlike traditional methods that require molds or stencils, this 3D printing assembly method can convert digital designs into complex 3D products causing little material wasting [8]. In addition, 3D printing technology makes the rapid manufacture of products possible [9], greatly advancing industrial production and academic study. Today, more than 50 kinds of 3D printing technologies based on different principles have been developed for different materials, speed, and precision requirements [10]. Due to the diversity of materials and printing methods [11], 3D printing technology has evolved into a universal and powerful technology for advanced manufacturing in the future platform [12]. Especially in soft polymer materials with various polymeric properties [13], the development of 3D printing technology has made it possible to directly construct complex functional soft systems [14].

Electromechanical engineering, chemical engineering, and sensor engineering are the foundations of the rapid development of soft robots [15,16,17]. However, high-level applications are usually inspired by biology [18,19]. Invertebrates of varying body complexity, from worms [20] to octopuses [21], provide inspiration in soft robot design. For example, humans have the feedback provided by the special receptors and afferent neurons that make up the somatosensory system, as well as the deformation, self-healing, and diversity-dependent dexterous hands and movements of animal muscle tissue and sensory network [22,23,24]. A new area of soft robotics seeks to replicate these features in a myriad of applications. This review mainly introduces recent progress in 3D-printing soft materials to build functional soft matters, especially those in biologically-related fields.

## 2. 3D Printing Method

### 2.1. Fused Deposition Modelling

**Figure 1:**
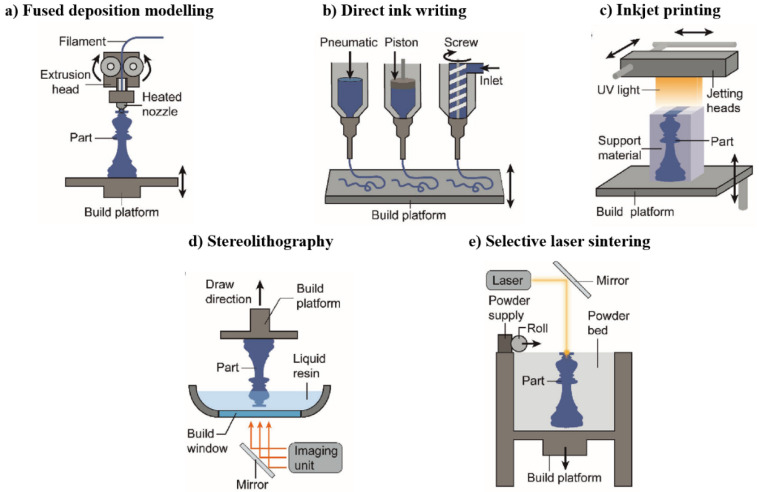
3D printing method. Reproduced with permission from ref. [27]. Copyright 2018, John Wiley and Sons.

Fused Deposition Modeling (FDM) additive manufacturing technology currently occupies about 6% of the 3D printer market, which is a promising printing method (Figure 1a). At the same time, FDM printers have high printing efficiency and no merits—polluting odor and little deformation after molding have gradually expanded the user group of FDM, which has wide commercial value. The FDM 3D printer consists of a wire extrusion device, a heating block, a nozzle, a printing platform, and a movement mechanism. Its molding process is based on a prefabricated shape, with the molten material in the form of a filament on the printing platform, and finally you obtain the target pattern which can be easily divided into four levels of dotted line and surface integration, which has obvious advantages over traditional 3D printing technology in the mold making process. FDM involves repeating the melting and cooling process, which limits its use in thermo-sensitive polymers. Soft robots made of thermoplastic polyurethane by the Ninjaflex series have been considered the most successful FMD fabrications, which can withstand strain γ_ult_ more than 500% and a Young’s modulus of 10 Mpa [25]. The nozzle diameter limited the resolution of FDM, and heterogeneity, defects, or voids can be introduced in the printing process. In addition, to avoid voids, the temperature must be controlled to make sure that the wire melts completely in the nozzle [26]. The construction time is directly proportional to the construction volume and inversely proportional to the resolution and nozzle size. FDM printers with particle hoppers or multiple nozzles can selectively deposit different kinds of thermoplastic materials in the same layer, paving the way for the complex mechanical programming required for multi-material printing and advanced robotic equipment.

### 2.2. Direct Ink Writing

Direct Ink Writing (DIW) is currently one of the most flexible 3D printing technologies available (Figure 1b). The basic principle is to precharge the ink material into the printhead and use the power source, such as an electric drive or high pressure gas, to power the vitality so that, even though the oil is extruded from the nozzle, the extruded ink is solidified through vulcanized [28], phase change [29], gelation [30], solvent evaporation [31], and other methods, then stacked layer by layer. With DIW technology, the rheological properties of the paint material determine the extrusion performance and shape accuracy to a certain extent, and the curing speed of the paint after extrusion also determines the properties of the horizontal structure and size.

### 2.3. Direct Inkjet Printing

Direct inkjet printing (Figure 1c) includes hot melt printing, powder bed inkjet printing, multi-inkjet printing, etc. The specific process is to spray the molten material onto the substrate before it hardens. The entire process is divided into three steps. The small water droplets form an ink jet (volume V = 1–100 pl and diameter, d = 10–150 μm) and are deposited directly on the substrate, which leads to the interaction between neighboring droplets, followed by the vitrified, vaporized, or polymerized ink solidifying. The thermosetting polymer droplets also contain photopolymers which are crosslinked when irradiated with ultraviolet light. Eventually, the material is accumulated through repeated production of two-dimensional layers.

### 2.4. Vat Photopolymerization

Reductive polymerization techniques, generally referred to as stereolithography (SLA) (Figure 1d), including double photon polymerization [32], micro-photolithography (μ-SL), digital mask projection three-dimensional photolithography [33], digital light treatment [34], and continuous liquid interface production [35], can be used to polymer in a liquid resin. The density medium provides a buoyancy that can support the soft and compliant structure, which can be printed and have a slope structure. The reduced polymerization printer produces selectively photocatalytic substantial layers by controlling the space and time of light. Different light sources are needed according to the difference in technology. A high resolution [35] is maintained while making a plurality of parts (stretching speeds to 1 m/h) at a rapid parallel resin system. Therefore, SLA provides an efficient and commercial technology for constructing soft robots with microscale features.

### 2.5. Selective Laser Sintering

Selective laser sintering (SLS) (Figure 1e) refers to building matters from powder particles. When printing, the laser rasterizes over the powder bed. The small particle melts as soon as the local temperature is above T_m_, and the materials cool down when the radiation stops. Then, the next layer of powder is applied along the print bed. The entire structure is built up as the process is repeated.

### 2.6. Summary

3D printing of soft polymer materials is a process of molding and curing in three-dimension space. Different 3D printing techniques work on different principles and materials, such as selective laser compaction and stereolithography, which selectively solidifies slices in a material tank based on the patterns. Ink-jet printing and extrusion printing move the material through the nozzle to the specified position, which is then cured. Therefore, a change in printing material can be achieved by changing the nozzle, which is difficult for SLS and SLA. Three-dimensional printing of soft materials mainly includes thermoplastic polymer, thermosetting polymer, photoinduced polymer, and physical cross-linked polymer. Thermoplastic polymers and photoinduced polymers are suitable for SLS and SLA. The nozzle-based printing method can print almost all materials with rheological properties and also has the ability of multi-material printing. SLS has significant advantages in printing large structures since it prints one layer at a time. Table 1 shows a comparison of these 3D printing methods. All in all, these 3D printing technologies have no absolute advantages and disadvantages, and appropriate printing methods should be selected according to actual requirements.

## 3. Applications of 3D Printed Soft Materials

### 3.1. Bio-Inspired Structures

The evolution of nature over millions of years to develop high-performance biological structures provides ideas for human design of high-strength and tough materials [42]. They typically consist of hard and soft phases arranged in complex hierarchies with feature sizes ranging from nanoscale to macroscale [43]. The resulting materials are lightweight and often exhibit unique combinations of strength and toughness, but they are difficult to imitate synthetically, thus hindering the development of biomimetic designs. The fabrication of biomimetic complex structures is a great challenge for the industry because it involves multiscale, multi-material binding, and multifunctional integration [44,45]. The rapid development of multi-material 3D printing technology in recent years provides a new solution to this problem [46,47].

**Figure 2:**
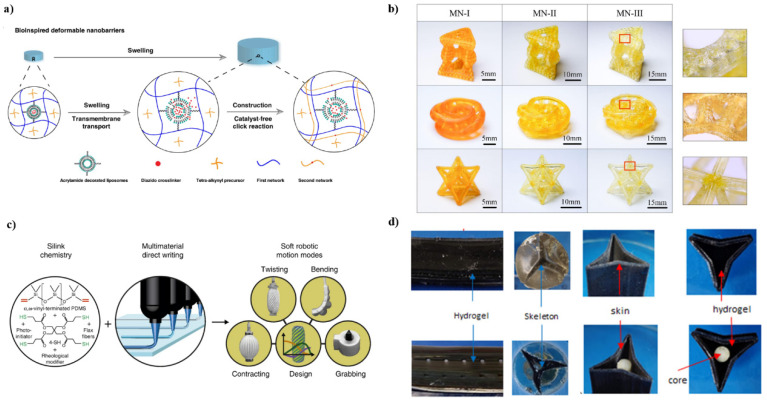
(**a**) Diffusion-inspired transport across synthetic liposome membranes by biological membranes. Reproduced with permission from ref. [53]. Copyright 2020, Springer Nature. (**b**) Self-grown multinetwork (MN) complex structures composed of Mobius shells, Kagome lattices, and octet lattices were fabricated by 3D printing. Reproduced with permission from ref. [54]. Copyright 2022, Elsevier. (**c**) Light-curable silicone inks have variable stiffness; multi-materials 3D printing with different stiffness in a single print with seamless combines can be obtained via precise programming. Reproduced with permission from ref. [55]. Copyright 2018, Springer Nature. (**d**) The left two columns are hydrogel–elastomer combined multi-material structures, and the right two columns are artificial cactus shapes. Reproduced with permission from ref. [56]. Copyright 1969, Elsevier.

When a synthetic material is molded into a particular shape, its dimensions and mechanical properties are permanently fixed. Structural shrinkage due to component extraction and structural expansion due to solvent addition often lead to weakening effects such as disintegration and destruction [48,49]. In contrast, natural living tissues, such as skeletal muscle, become stronger with a continuous supply of water and amino acids after repeated growth cycles, forming new components within the original tissue [50,51]. The researchers exploited this self-growth and self-reinforcing phenomenon to design smart materials with dynamic and programmable properties. Scholars in Japan used mechanical free radicals generated by chain scission to achieve repairable and strengthened double-network hydrogels [52]. Adding additional monomers to a partially damaged hydrogel can form a new polymer network, improving strength and toughness. Wu et.al. encapsulated dispersed diazide-based crosslinkers in a single hydrogel network with a deformable barrier [53]. After swelling in water, the cross-linking agent is released from the capsule, forming an additional second network that increases the strength and modulus of the hydrogel (Figure 2a). Thus far, self-grown materials with simple shapes have been developed, and how to break through the limitations of network types and enhancers is the key. Drawing on the continuous biological self-growth process, Wu et.al. proposed a solvent-free photocurable elastomer system which successfully fabricated high-precision and high-complexity shapes [54]. Self-growth of printed structures can be achieved without changing the chemical structure by sequentially dipping the structures into the same type of monomer without the addition of a crosslinking agent (Figure 2b). On-demand enhancement of the modulus and strength of the printed structure can be obtained with an adjustable growth cycle. Due to changes in network stretch during the growth cycle, 3D printed multinetwork (MN) can also be used as a waterproof structure.

Manuel Schaffner et.al. reported a 3D printing platform for seamless digital manufacturing of pneumatic silicone actuators with programmable biomimetic structures and movements [55]. The actuator is made of elastomer and its surface is decorated with reinforcing strips with clear chamfers. Similar to the fiber structure of muscle hydrostats, the lead angle can be varied to achieve elongation, contraction, or twisting motion (Figure 2c). The design principles for digital fabrication of silicon-based soft actuators are based on a quantitative model of stacking theory. The functional response of the material can be programmed based on its properties and structure. By exploring this programmable potential, 3D printing of various soft deformable structures will be possible.

The two developmental models (Figure 2d) explored by Anil K. Bastola et al. [56] suggest that there is much to learn about plant ecology, development, and adaptive behavior. These biological systems provide a ready source of information for designing artificial systems with developmental intelligence and adaptability. This controllable movement (bending) and shape change can be used to monitor, for example, changes in humidity and to develop humidity-based actuators for plant-inspired ecorobotic systems. Anil K. Bastola et al. proposed multiple soft robotic schemes based on the growth and development of a climbing cactus, Selenicereus setaceus, in the seasonally arid Brazilian Atlantic Forest. In its natural habitat, the cactus stem develops striking changes in cross-sectional geometry, adaptively performing different functional roles in response to external cues and environmental constraints. Anil K. Bastola et al. demonstrated the inspiration and cactus-based structural configuration of a multi-material, hydrogel-elastomer, and biphasic soft robotic system.

### 3.2. Soft Robots

**Figure 3:**
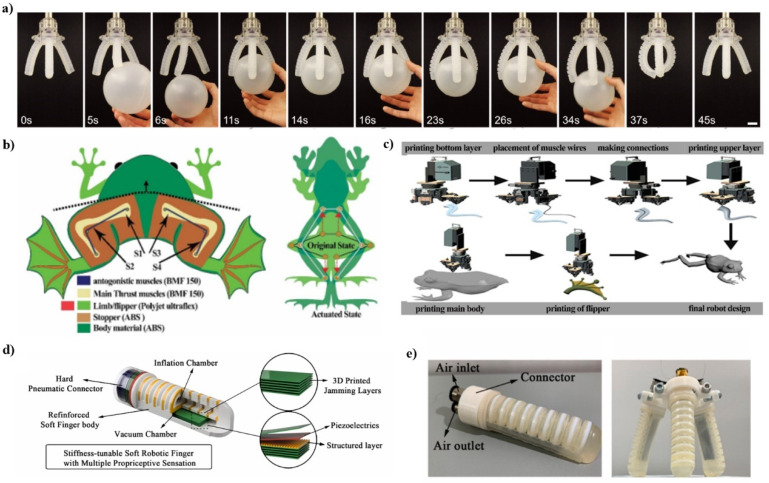
(**a**) Images of an interaction process between a ball and a soft robotic gripper comprised of SSAs. Reproduced with permission from ref. [57]. Copyright 2018, John Wiley and Sons. (**b**) Schematic diagram of the working mechanism; the left image shows the dual method of generating thrust, and the right image shows synchronized swimming. Reproduced with permission from ref. [59]. Copyright 2021, Elsevier. (**c**) EXPOG manufacturing process flow diagram. A fully functional frog robot is printed and assembled step by step. First, one half of the limb was printed with ultraflex filament, the second joint was used to place the muscle wires pre-soldered for connection, then the second half of the limb and flippers were printed of the same material, while the predecessor robot was printed with ABS filament, and finally all the parts were printed connected. Reproduced with permission from ref. [59]. Copyright 2021, Elsevier. (**d**) Schematic of the soft robotic finger. (**e**) The three-finger gripper for practical grasping. Reproduced with permission from ref. [58]. Copyright 2021, Elsevier.

The somatosensory system provides feedback for humans to achieve manual dexterity and control of various movements of the human body. Ryan L. Truby et.al. report on how to create soft body-sensitive actuators (SSAs) via embedded 3D printing that are controlled by multiple conductive functions and enable tactile, proprioceptive, and thermal sensations at the same time [57]. This new manufacturing method seamlessly integrates multiple ionic and fluid features into the elastic matrix to produce SSAs with the desired biomimetic sensing and working functions. Each printed sensor consists of an ionic conductive gel that provides long-term stability and hysteresis-free performance. For example, combining multiple SSAs with a soft robot gripper provides proprioceptive and tactile feedback through embedded curvature, expansion, and contact sensors such as depth and fine touch contact sensors (Figure 3a). The multi-material manufacturing platform makes it easy to integrate complex sensing patterns into soft actuation system. Xie et al. fabricated a soft finger robot with adjustable stiffness, which can work without an external power [58]. Figure 3d,e shows the schematic of this soft finger robot with low power sensor which can provide sense and energy absorption, and thus can interact with the environment safely. By way of a properly designed sensor, a tunable-stiffness soft robot can have the ability to interact with objects. This is a necessary step to achieve closed-loop feedback control for soft robots, machines, and haptic devices.

Afaque Manzoor Soomro et al. [59], inspired by the relatively simple morphology of aneurids such as Rana Esculenta (a semi-aquatic frog), proposed a shape memory alloy (SMA)-based multilayer structure design and ultra-flexible material for the design, fabrication, and characterization of a flexible biomimetic robotic frog. A dual-thrust generation method using four SMA myofilaments to realize synchronous swimming of frogs was proposed. The frog robot, named “Exploring Frog”, is made with a multi-head 3D printing system. The robot is designed based on mathematical modeling, simulation, and fluid dynamics analysis of real frogs. This soft biological frog robot (EXPOG) is able to swim synchronously underwater.

First, the synchronous swimming motion of frogs was analyzed by TRACKER software. Subsequently, the model was proposed with the motion control equation as the focus, and an in-depth theoretical analysis was carried out (Figure 3b). In addition, Fluidic-Solid Interactions (FSI) simulations were performed in COMSOL to verify the design of the frog stroke, the surface velocity, and the generation of vortices in the water. Based on the model and simulation results, a multi-head 3D printer was used to make the robot (Figure 3c).

### 3.3. Flexible Electronics

**Figure 4:**
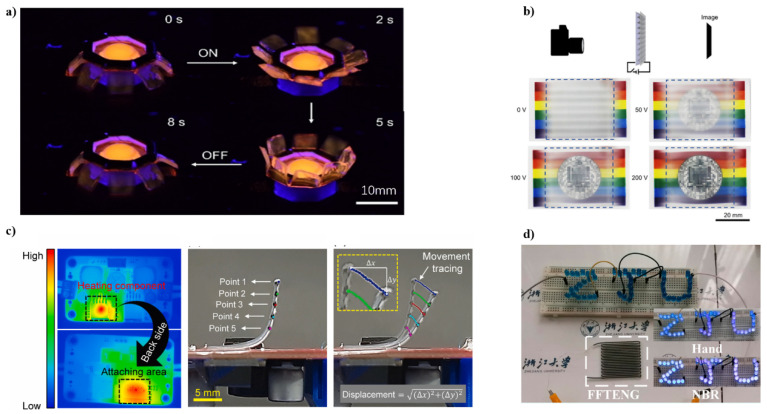
(**a**) Photograph of a printed PVC gel jellyfish actuator with 1000 V applied. Add the fluorescent dye Rhodamine B to the ink for visualization. Photos were taken in dark conditions and 365 nm UV light. Reproduced with permission from ref. [63]. Copyright 2021, American Chemical Society. (**b**) Voltage can change image transparency. When the applied voltage was increased from 0 V to 200 V, the image behind the PVC gel-based smart window changed from completely opaque to transparent. Reproduced with permission from ref. [63]. Copyright 2021, American Chemical Society. (**c**) Temperature distribution of the electrical DC-DC converter module (shown on the left) The middle image shows five points on the side of the actuator, spaced 2 mm apart. The image on the right shows real-time five-point motion tracking to estimate the position of the actuator at a specific time during the heating process. Reproduced with permission from ref. [64]. Copyright 2021, Elsevier. (**d**) Self-power LED when tapped by bare hand and a NBR-covered hand. Reproduced with permission from ref. [65]. Copyright 2021, Elsevier.

The rapid development of 3D printing has provided a new technique for fabricating flexible electronics, which enables the application of conductive biomaterial. The design of bio-ink with printability, conductivity, and that is harmless to the body is crucial for bio-electronics [60,61,62]. Laminated modeling of electrically responsive soft actuators has important implications for the design and construction of new soft robots and machines. However, the options for soft materials that are 3D printable and electrically responsive are very limited. Wang et.al. report an electrically controllable 3D printing strategy for polyvinyl chloride (PVC) gel actuators [63]. An actuator similar to a jellyfish is printed with PVC ink (Figure 4a) and can be bent 130° in less than 5 s. As a proof-of-concept demonstration, a 3D-printed PVC gel-based smart window will show that its transparency can be changed when a voltage is applied (Figure 4b). The 3D printing strategy developed in this paper has the potential to expand the potential use of electrically responsive soft materials in a variety of engineering disciplines.

Sanghun Shin et al. succeeded in manufacturing a heat-responsive soft actuator using 3D printing technology, demonstrating its application as a switch for electrical applications [64]. PLA filaments are printed directly on commercially available paper (paperboard) to form a two-layer composite structure. This will program the reversible actuator. PLA is not soft at room temperature due to its high hardness, but it is soft and elastic due to thermal stimulation exceeding the glass transition temperature (Figure 4c). Finally, a simple additional electrical connection allows the device to provide a time interval signal in response to stable heat transfer. During operation, the manufactured switch is connected to the heat source (heating component) of a commercially available DC-DC converter module. The system consists of two switches; the distance between the 3-STA and the pin header controls the time gap and provides step input voltage to other equipment, including the cooling system. Therefore, the wind speed (or flow rate) of the cooling fan can be adjusted according to the bottom temperature of the switch. Simply put, when the electronics under the switch become hot, the system supplies more power and provides more airflow for proper cooling. Sanghun Shin et al. demonstrate efficient/easy manufacturing and transient analysis of electrical switches consisting of soft polymer actuators for electrical applications.

A fully flexible single-electrode TENG (FFTENG) was fabricated by Wang et al. via direct ink writing 3D printing method with complex pattern [65]. A silicone elastomer shell is used as the triboelectric layer and an inner silicone/carbon black (CB) core as the flexible electrode. Figure 4d shows this self-power LED, which was lighted by continuously tapping the FFTENG using a bare hand and an NBR-covered hand. The electron affinity of NBR is lower than bare skin, thus, when tapped by the NBR-covered hand, the LED is brighter.

Due to the flexibility of 3D printing methods, various flexible electronics can be fabricated to meet actual need such as LED sensors, actuators, and switch, which offers a useful solution for next generation human–machine interaction device.

### 3.4. Biomedical Engineering

**Figure 5:**
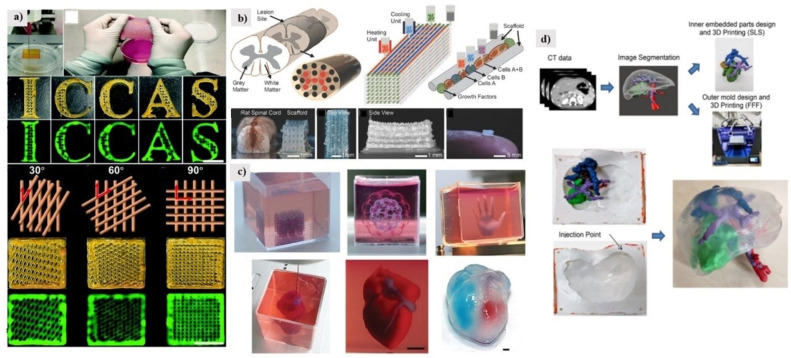
(**a**) 3D architectures printed from the Gel/Alg/HA/PPV ink. Reproduced with permission from ref. [69]. Copyright 2022, Royal Society of Chemistry. (**b**) Experimental strategies for 3D bioprinting spinal cord tissue. Reproduced with permission from ref. [70]. Copyright 2018, John Wiley and Sons. (**c**) Printing of personalized hydrogels in support media. Reproduced with permission from ref. [71]. Copyright 2019, John Wiley and Sons. (**d**) Model design workflow and 3D-produced surgical plan prototype. Reproduced with permission from ref. [72]. Copyright 2020, Elsevier.

3D bio-printing offers an advanced method for fabricating living tissues, which will change the field of surgery [66]. Bio-materials science, mechanical science, and surgical science are the basic elements of nature. Having a good command of producing tissue in three-dimensions is the critical basis for regenerative treatment [67,68]. Inspired by the biological function of the skin to protect the body from the invasion of microorganisms, the artificial creation of human skin with anti-infection and skin regeneration capabilities in vitro is an urgent need for wound repair. Zhan et al. developed printable inks composed of natural biopolymers such as gelatin (Gel), alginate (Alg), hyaluronic acid (HA), and photoactive cationic conjugated polyphenylene vinyl derivatives (PPV) to make a 3D printed artificial skin patch [69]. Compared to other dressings based on the hydrogel system, the skin patch developed by Zhan et al. has comprehensive antibacterial ability, tissue regeneration promoting ability, and abundant microstructural patterns, and was suitable as a skin equivalent when a skin trauma occurred (Figure 5a).

By assembling induced pluripotent stem cell (iPSC)-derived spinal neuronal progenitor cells (sNPCs) and oligodendrocyte progenitor cells (OPCs), a bioengineered spinal cord fabricated by multi-material 3D bioprinting can be achieved, placed at the precise location of the 3D printed biocompatible scaffold (Figure 5b). Daeha Joung et al. created a 3D spinal cord tissue-like platform via a one-pot 3D bioprinting method involving neuronal and glial progenitor cells in a biocompatible scaffold [70]. Daeha Joung et al. created fully 3D bioprinted neural progenitor cells with axonal propagation in engineered 3D biocompatible scaffolds.

In cardiac tissue engineering, generating thick vascularized tissue that perfectly matches the patient remains an unmet challenge. Nadav Noor et al. report a simple method to 3D print thick, vascularized, perfusable cardiac patches that fully conform to the patient’s immune, cellular, biochemical, and anatomical properties [71]. To do this, biopsies of omental tissue are taken from the patient. An artificial heart with cellularization was successfully printed, verifying the feasibility of cellular programming. The extracellular matrix consisted of personalized hydrogels and was bound to cardiomyocytes and endothelial cells to form bio-inks for cardiac parenchyma tissue and blood vessels, respectively, as shown in Figure 5c.

In the study of A. Tejo-Otero et al. [72], liver tissue was simulated and a prototype surgical plan was fabricated (Figure 5d). Surgeons can rehearse it and confirm its benefits in preoperative surgical planning. This liver phantom can be used for medical school and patient education in addition to preoperative surgical planning. The latest results are still far from in vitro organ functional tissue/organ reconstruction, but advances in 3D printing and bioprinting technology have brought this goal one step closer and made the impossible achievable. We summarized the applications of 3D printing soft matters, as shown in Table 2.

## 4. Conclusions

To sum up, remarkable progress has been made in 3D printing soft matters which are inspired by natural beings. However, as an emerging field, there are still many challenges in terms of equipment, materials, and design. This paper summarizes the 3D printing technology of soft matters and the application of its products in the fields of bionics, soft robotics, electronic sensing, and biomedical engineering. An effective method to fabricate complex functional soft matters is by using multi-material 3D printing technology. In order to improve the integration and function of the printed structures, advanced materials, equipment, and computer technology play an important role, which would surely benefit the 3D printing soft materials technology and thus improve human beings’ lives in the near future.

## Figures and Tables

**Table 1:** Comparison of different 3D printing methods.

Methods	Resolution	Relative Build Speed ▲	Raw Materials	Multi-Material Printing Ability ▲
Fused deposition modelling	>100 μm [36]	▲	thermoplastic polymers	▲▲
Direct ink writing	1~100 μm [37]	▲	Curable pseudoplastic polymer fluids	▲▲▲
Direct ink printing	>10 μm [38]	▲▲	Low viscosity polymer fluids	▲▲▲
Stereolithography	>5 μm [39,40]	▲▲	Photopolymers with low viscosity	▲▲
Selective laser sintering	>100 μm [41]	▲▲▲	Thermoplastic polymers	▲

**Table 2:** Summary of 3D printing applications.

Application	Material	Method	Key Point	Advantage
Bio-inspired structure	3D printable resin [54]	Digital light processing 3D printing	Solvent-free elastomer composite system	Self-growing composites
	Light-curable silicone inks [55]	DIW	Alike plant systems and muscular hydrostats	Programmable
	TangoPlus [56]	Object260 3D printer	Multi-material biphasic soft system	Does not require additional sources of energy
Soft robots	Conductive ionogel and fugitive inks [57]	DIW	Embedded 3D printing	Emulate the human somatosensory system
	Multi-material [58]	Objet350 3D printing	Built-in multifunctional sensor	Self-powered, flexible multifunctional sensor
	ABS, ultraflex [59]	Custom-made multiheaded 3D printing system	Multilayer structural design	Synchronous swimming of frog
Flexible electronics	PVC ink [63]	DIW	Triggered by an electric field	A facile way to print PVC gel actuators
	PLA [64]	FDM-based 3D printer	A bilayer composite	Without any complicated control systems
	Silicone/carbon black 3D printing ink [65]	Coaxial DIW 3D printing	Fully flexible single-electrode TENG	Convert biomechanical energy into electric energy
Biomedical engineering	Phenylene vinylene and gelatin/alginate/hyaluronic acid ink [69]	A commercial 3D printer	Ink design principle	Dual biofunctions of anti-infection and promoting soft tissue regeneration
	Bio-inks [71]	A 3D printer, equipped with extrusion-based print heads	Bio-inks originated from the same patient	Fully match any individual
	Polyamide, poly lactic acid [72]	Selective laser sintering and fused filament fabrication	Viscoelasticity and hardness	Allow the different anatomical structures to be replicated

## Data Availability

Not applicable.
